# Hospital Readmission Due to Chronic Obstructive Pulmonary Disease: A Longitudinal Study

**DOI:** 10.34172/ijhpm.2022.5770

**Published:** 2022-01-09

**Authors:** Chidiamara Maria Njoku, Barbara Caecilia Wimmer, Gregory Mark Peterson, Leigh Kinsman, Bonnie Jayne Bereznicki

**Affiliations:** ^1^School of Pharmacy and Pharmacology, College of Health and Medicine, University of Tasmania, Hobart, TAS, Australia.; ^2^School of Nursing and Midwifery, University of Newcastle, Port Macquarie, NSW, Australia.; ^3^Tasmanian School of Medicine, College of Health and Medicine, University of Tasmania, Hobart, TAS, Australia.

**Keywords:** COPD, Patient Readmission, Prevalence, Risk Factors

## Abstract

**Background:** This study aimed to investigate the prevalence of hospital readmission for chronic obstructive pulmonary disease (COPD) at 30, 90 and 365 days, and to determine demographic and socioeconomic risk factors for 30-day and 90-day readmission and time to COPD-related readmission within 365 days in Tasmania.

**Methods:** Patients ≥40 years admitted for COPD between 2011 and 2015 were identified using administrative data from all major public hospitals in Tasmania, Australia. Factors associated with readmission and time to readmission were identified using logistic and Cox regression, respectively.

**Results:** The rates of COPD-related readmission were 6.7% within 30 days, 12.2% within 90 days and 23.7% within 365 days. Being male (odds ratio [OR]: 1.49, CI: 1.06–2.09), Indigenous (OR: 2.47, CI: 1.31–4.66) and living in the lower socioeconomic North-West region of Tasmania (OR: 1.80, CI: 1.20–2.69) were risk factors for 30-day readmission. Increased COPD-related (OR: 1.48, CI: 1.22–1.80; OR: 1.52, CI: 1.29–1.78) and non-COPD-related (OR: 1.12, CI: 1.03– 1.23; OR: 1.11, CI: 1.03–1.21) emergency department (ED) visits in the preceding six months were risk factors for both 30-day and 90-day readmissions. Being Indigenous (hazard ratio [HR]: 1.61, CI: 1.10–2.37) and previous COPD-related ED visits (HR: 1.30, CI: 1.21–1.39) decreased, while a higher Charlson Comorbidity Index (CCI) (OR: 0.91, CI: 0.83– 0.99) increased the time to readmission within 365 days.

**Conclusion:** Being male, Indigenous, living in the North-West region and previous ED visits were associated with increased risk of COPD readmission in Tasmania. Interventions to improve access to primary healthcare for these groups may reduce COPD-related readmissions.

## Background

**Key Messages**

** Implications for policy makers**

- Being male, Indigenous, having recent emergency department (ED) visit and living in the lower socioeconomic region of Tasmania were significant demographic and socioeconomic predictors of chronic obstructive pulmonary disease (COPD)-related readmission.
- Improving patient-centred access to healthcare within the community, especially for Indigenous people, male patients and those living in lower socioeconomic areas, may reduce COPD-related readmissions.
- Future studies focused on improving patient-centred access to healthcare within the community and providing community support framework may divert mild and moderate cases of COPD from hospital.

** Implications for the public**

Hospital readmission for chronic obstructive pulmonary disease (COPD) places a substantial burden on patients and healthcare systems around the globe. In the present study, we aimed to investigate the prevalence of hospital readmission for COPD at 30, 90, and 365 days and to determine the risk factors for 30-day and 90-day readmission and time to COPD-related readmission within 365 days. Our findings indicated that male patients, Indigenous people, and those living in the lower socioeconomic region were more likely to be readmitted for COPD in Tasmania. Sustainable interventions (eg, smoking cessation, education) directed at Indigenous people, and those living in the lower socioeconomic region of Tasmania may avert and lessen COPD-related readmission.

Chronic obstructive pulmonary disease (COPD) places a substantial burden on patients and healthcare systems around the globe. COPD is the third leading cause of death, accounting for 4.7 million annual deaths worldwide.^1,2^ It was the fifth leading cause of disability-adjusted life years lost across the world in 2013.^3^ Based on the Global Initiative for Obstructive Lung Disease criteria, 14.5% of Australians aged 40 years and over had COPD in 2010.^4^ COPD was the most common chronic condition associated with potentially preventable hospitalisation in four of the eight states and territories of Australia in 2015-2016.^5^ Tasmania is the only Australian island state, with a population of 530 000,^6^ predominantly regional and rural.^7^ In Tasmania, COPD was the third leading cause of potentially preventable hospitalisation in 2015-2016, with 218 admissions per 100 000 population, which increased by 9.0% in 2016-2017.^8^

Previous research from the United States showed that 20% of patients admitted with an exacerbation of COPD were readmitted within 30 days,^9^ and 50.0% were readmitted within six months^10^ of hospital discharge. A recent systematic review highlighted inconsistencies across several risk factors previously reported as being associated with readmission for COPD.^11^ This review demonstrated that hospitalisation in the previous year was the key predictor of readmission.^11^ Patients living in deprived areas and in nursing homes were also at increased risk of readmission for COPD. However, there were variations in the reported factors associated with risk of readmission, which may reflect differences in the local context, such as the availability of community-based services to prevent and care for exacerbation of COPD. It was recommended that risk factors for COPD-related readmissions should be considered in the light of locality due to variations in healthcare systems around the world.^11^

A few Australian studies have addressed risk factors for all-cause readmission following a COPD index admission,^12,13^ but none have explored how patient characteristics relate to readmissions specifically for COPD. As an island state, Tasmania has minimal interstate hospital visits for patients in the community, making it an ideal setting for longitudinal research. This study aimed to measure the prevalence of 30-day, 90-day and 365-day COPD-related readmission in Tasmania. Additional aims were to identify the risk factors associated with 30-day and 90-day COPD-related readmission, and time to readmission within 365-days.

## Methods

###  Study Design and Setting

In this longitudinal study, a retrospective cohort of patients admitted with COPD to any of the four main public hospitals in Tasmania between January 1, 2011 and December 31, 2015 were reviewed. Data were collected until December 31, 2016, to allow 365 days follow-up for each patient. These hospitals (Royal Hobart Hospital, Launceston General Hospital, North West Regional Hospital and Mersey Community Hospital) collectively account for 95% of all public hospital admissions.^14^ The population of Tasmania is dispersed across three regions: Hobart (South; 271 214 persons), Launceston (North; 145 033 persons) and the North-West Coast (111 954 persons), where these hospitals are located.^6^ Socioeconomic status across the three regions varies. Within the socioeconomic ranking across Australia, Southern Tasmania ranks among the highest, followed by the North, with the North-West ranking lowest.^15^ Four percent of Tasmanian residents identfiy as Indigenous.^16^

###  Data Source

 The study utilised the anonymised administrative admitted patient care National Minimum Data Set from the Department of Health and Human Services, Tasmania. The dataset provided access to de-identified demographic, administrative and clinical information pertaining to all COPD-related admissions and readmissions from January 1, 2011 to December 31, 2016. Mortality records of admitted patients were linked to the dataset.

###  Study Participants

 The study comprised patients aged 40 years and over, who had an overnight hospitalisation with a primary diagnosis of COPD between January 1, 2011 and December 31, 2015 (index admission). The diagnosis of COPD was determined using the International Statistical Classification of Diseases and Related Health Problems, Tenth Revision, Australian Modification (ICD-10-AM) codes (J40–J44).

###  Measures

 All patients with an index admission were followed up for 365 days post-index discharge, except in the case of death occurring first. COPD-related readmissions following the index admission within 30, 90, and 365 days were analysed for all patients. COPD-related readmissions were subsequent admissions with a primary diagnosis of COPD. Non-readmitted patients were those who had an index admission but did not have a subsequent COPD-related readmission within the follow-up period. Those who died were identified from the mortality records that were linked to the dataset.

Based on well-established evidence on the relationship between previous emergency department (ED) visits and COPD-related readmission, the numbers of COPD-related and non-COPD-related ED visits in the six months preceding the index admission were both recorded as covariates.^11^ Socioeconomic status of patients was estimated from their residential address using the Index of Relative Socio-economic Advantage and Disadvantage (IRSAD) for areas.^17^ Based on the Australian Bureau of Statistics recommendation, deciles were used in ranking the index of IRSAD.^17^ Patients’ usual residence was also used to group participants into the three main geographical regions (South, North and North-West) in Tasmania. Other covariates that were considered included age at index admission, sex, country of birth (Australia or overseas) and Indigenous status. Variables related to patients’ index admission included discharge destination, admission to intensive care, length of stay, weekday or weekend admission and season of admission. The Charlson Comorbidity Index (CCI) ^18^ was used to determine the level of comorbidity.

###  Statistical Analysis

 Data were analysed using STATA version 16.1 (StataCorp LLC, College Station, TX, USA). Descriptive analyses were used to report the prevalence of COPD-related readmission. Demographic and clinical characteristics of patients who were and were not readmitted during the follow-up periods were compared. Chi-square tests were used to compare categorical variables, and Mann-Whitney U-tests were used to compare continuous variables, between patients who were and were not readmitted.

Multiple logistic regression was performed to determine the factors independently associated with 30-day and 90-day readmission. Based on previous studies,^11^ age and sex, together with variables with *P* values of ≤.10 in the univariate analyses, qualified for entry into the logistic regression models. Prior to performing logistic regression, variables were tested for inter-correlation. Categorical variables were assessed for collinearity against continuous variables using the Mann-Whitney U test and included in the logistic regression model if *P* ≥ .05. Continuous or ordinal variables were assessed for collinearity against other continuous variables using a correlation test and included if the Spearman rho <0.40.

Cox proportional hazards regression was used to identify independent factors associated with time to first COPD readmission within 365 days. Bivariate analyses (ie, simple Cox regression) were performed for each variable. Age, gender and variables with *P* values of ≤.10 were included in the multiple Cox proportional hazard regression. The end of follow-up was set at 365 days after hospital discharge or date of death, whichever occurred first. Proportional hazards assumption was assessed for each variable by visual inspection of the log-minus-log plots across the covariate categories and by analysing residuals. Proportional hazard assumptions were confirmed using the test for parallel lines.

## Results

###  Prevalence of COPD-Related Readmission in Tasmania

Between January 1, 2011 and December 31, 2015, there were 2448 patients ≥40 years who had an overnight index admission with COPD as the primary diagnosis and were followed up for subsequent COPD-related readmissions (Table 1). The rate of COPD-related readmission was 6.7% within 30 days, 12.2% within 90 days and 23.7% within 365 days (Figure).

**Table 1:** Demographic and Admission Characteristics of All Patients With Chronic Obstructive Pulmonary Disease Index Admission

**Characteristics**	**All Patients (n = 2448)**
Age (y), median (IQR)	72 (64–80)
Gender, No. (%)	
Male	1222 (49.9)
Female	1226 (50.1)
Country of birth, No. (%)	
Australian born	2150 (87.8)
Overseas born	298 (12.2)
Indigenous status, No. (%)	
Indigenous	97 (4.0)
Non-Indigenous	2340 (95.6)
Missing data	11 (0.4)
Tasmanian region, No. (%)	
South	991 (40.5)
North	696 (28.4)
North-West	724 (29.6)
Interstate patients	37 (1.5)
IRSAD decile	3 (2–5)
CCI, median (IQR)	0 (0–1)
Length of index stay (days), median (IQR)	3.9 (2.1–6.8)
Intensive care admission at index, No. (%)
Yes	141 (5.8)
No	2307 (94.2)
Discharge destination, No. (%)	
Home	2158 (88.2)
Nursing home	50 (2.0)
Other^a^	240 (9.8)
Admission day of the week, No. (%)	
Weekend	638 (26.1)
Weekday	1810 (73.9)
Australian season of admission, No. (%)	
Spring	684 (27.9)
Summer	489 (20.0)
Autumn	469 (19.2)
Winter	806 (32.9)

Abbreviations: IQR, interquartile range; CCI, Charlson Comorbidity Index; IRSAD, Index of Relative Socioeconomic Advantage and Disadvantage. ^a^Other: welfare institutions, prisons, mental and rehabilitation centres, private and rural hospitals.

**Figure:**
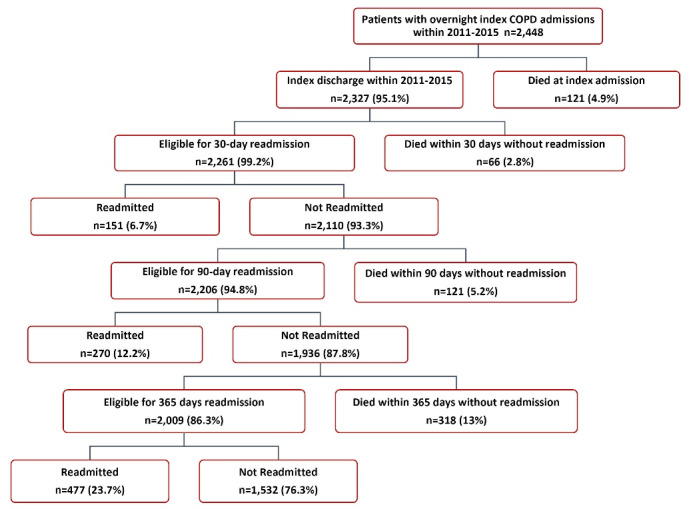
Prevalence Of 30-Day, 90-Day and 365-Day COPD Readmission in Tasmanian Public Hospitals. Abbreviation: COPD, chronic obstructive pulmonary disease.

###  Factors Associated With COPD-Related Readmissions

Table 2 shows the factors associated with 30-day, 90-day and 365-day readmissions. Six variables were included in the 30-day and the 90-day readmission multivariate models (Table 3). There was significant intercorrelation between Tasmanian region and IRSAD deciles (Kruskal-Wallis H test χ^2^ = 106.68, *P* = .0001), which resulted in the exclusion of IRSAD decile from the multivariate analyses.

 Being male (odds ratio [OR]: 1.49, 95% CI: 1.06–2.09), Indigenous (OR: 2.47, 95% CI: 1.31–4.66), living in the North-West region (OR: 1.80, 95% CI: 1.20–2.69), and increased COPD-related (OR: 1.48, 95% CI: 1.22–1.80) and non-COPD-related (OR: 1.12, 95% CI: 1.03–1.23) ED visits in the previous six months were significant risk factors for 30-day readmission. Increased COPD-related (OR: 1.52, 95% CI: 1.29–1.78) and non-COPD-related (OR: 1.11, 95% CI: 1.03–1.21) ED visits in the previous six months significantly increased the risk of 90-day readmission. The most common reasons for non-COPD related ED visits were pneumonia (1614/15 421; 10.5%), pain in the throat and chest (881/15 421; 5.7%) and heart failure (714/15 421; 4.6%).

**Table 2:** Demographic and Admission Characteristics Based on Readmission Status

**Characteristics**	**30-Day Readmission**				**90-Day Readmission**				**365-Day Readmission**			
**Variables**	**Total (n = 2261)**	**Yes (n = 151)**	**No (n = 2110)**	***P***	**Total (n = 2206)**	**Yes (n = 270)**	**No (n = 1936)**	***P***	**Total (n = 2009)**	**Yes (n = 477)**	**No (n = 1532)**	***P***
Age (y), median (IQR)	72 (64–80)	72 (64–79)	72 (64–80)	.28	72 (64–80)	73 (65–80)	72 (64–80)	.76	71 (64–79)	72 (64–79)	71 (64–79)	.91
Gender, n (%)												
Male	1117 (49.4)	89 (8.0)	1028 (92.0)	**.02**	1083 (49.1)	148 (13.7)	935 (86.3)	**.05**	968 (48.2)	238 (24.6)	730 (75.4)	.39
Female	1144 (50.6)	62 (5.4)	1082 (94.6)		1123 (50.9)	122 (10.9)	1001 (89.1)		**1041 (51.8)**	**239 (23.0)**	**802 (77.0)**	
Country of birth, n (%)												
Australian born	1989 (88.0)	137 (6.9)	1852 (93.1)	.28	1939 (87.9)	235 (12.1)	1704 (87.9)	.64	1768 (88.0)	419 (23.7)	1349 (76.3)	.90
Overseas born	272 (12.0)	14 (5.1)	258 (94.9)		267 (12.1)	35 (13.1)	232 (86.9)		241 (12.0)	58 (24.1)	183 (75.9)	
Indigenous status, n (%)												
Indigenous	89 (4.0)	14 (15.7)	75 (84.3)	**<.01**	88 (4.0)	16 (18.2)	72 (81.8)	.08	82 (4.1)	32 (39.0)	50 (61.0)	**<.01**
Non-Indigenous	2161 (95.6)	137 (6.3)	2024 (93.7)		2107 (95.5)	253 (12.0)	1854 (88.0)		1917 (95.4)	443 (23.1)	1474 (76.9)	
Missing data	11 (0.4)				11 (0.5)				11 (0.5)			
Tasmanian region, n (%)												
South	906 (40.1)	48 (5.3)	858 (94.7)	**.01**	889 (40.9)	108 (12.1)	781 (87.9)	.09	821 (40.9)	200 (24.4)	621 (75.6)	.55
North	651 (28.8)	42 (6.5)	609 (93.5)		625 (28.8)	66 (10.6)	559 (89.4)		558 (28.2)	126 (22.6)	432 (77.4)	
North-West	671 (29.7)	61 (9.1)	610 (90.9)		659 (30.3)	96 (14.6)	563 (85.4)		597 (30.2)	151 (25.3)	446 (74.7)	
Interstate patients	33 (1.5)				33 (1.5)				33 (1.6)			
IRSAD decile, median (IQR)	3 (2–5)	3 (2–4)	3 (2–5)	.26	3 (2–5)	3 (2–4)	3 (2–5)	**.02**	**3 (2–5)**	**3 (2-4)**	**3 (2–5)**	**.13**
CCI, median (IQR)	1 (1–2)	1 (1–2)	1 (1–2)	.74	1 (1–2)	1 (1–2)	1 (1–2)	.38	0 (0–1)	0 (0–1)	0 (0–1)	**.01**
Length of index stay (days), median (IQR)	4 (2–7)	4 (2–7)	4 (2–7)	.65	4 (2–6)	4 (2–7)	4 (2–6)	.33	4 (2,6)	4 (2,7)	4 (2,6)	**<.01**
Intensive care admission at index, n (%)												
Yes	111 (4.9)	5 (4.5)	106 (95.5)	.35	109 (4.9)	8 (7.3)	101 (92.7)	.11	95 (4.7)	21 (22.1)	74 (77.9)	.70
No	2150 (95.1)	146 (6.8)	2004 (93.2)		2097 (95.1)	262 (12.5)	1835 (87.5)		1914 (95.3)	456 (23.8)	1458 (76.2)	
Discharge destination, n (%)												
Home	2043 (90.4)	135 (6.6)	1908 (93.4)	.92	1998 (90.6)	246 (12.3)	1752 (87.7)	.90	1,832 (91.2)	439 (24.0)	1,393 (76.0)	.32
Nursing home	42 (1.9)	3 (7.1)	39 (92.9)		40 (1.8)	4 (10.0)	36 (90.0)		32 (1.6)	4 (12.5)	28 (87.5)	
Other^a^	176 (7.8)	13 (7.4)	163 (92.6)		168 (7.6)	20 (11.9)	148 (88.1)		145 (7.2)	34 (23.4)	111 (76.6)	
Number of ED visits in previous 6 months, median (IQR)												
COPD-related	1 (0–1)	1 (0–1)	1 (0–1)	**<.01**	1 (0–1)	1 (0–1)	1 (0–1)	**<.01**	1 (0,1)	0 (0,1)	0 (0,1)	**<.01**
Non-COPD-related	1 (0–1)	1 (0–2)	1 (0–1)	**.02**	1 (0–1)	1 (0–2)	1 (0–1)	**.02**	1 (0–1)	1 (0–2)	1 (0–1)	**.02**
Admission day of the week, n (%)												
Weekend	589 (26.1)	38 (6.5)	551 (93.5)	.80	576 (26.1)	68 (11.8)	508 (88.2)	.71	522 (26.0)	120 (23.0)	402 (77.0)	.64
Weekday	1672 (73.9)	113 (6.8)	1559 (93.2)		1630 (73.9)	202 (12.4)	1428 (87.6)		1487 (74.0)	357 (24.0)	1130 (76.0)	
Australian season of admission, n (%)												
Spring	620 (27.4)	42 (6.8)	578 (93.2)	.99	607 (27.5)	70 (11.5)	537 (88.5)	.13	557 (27.7)	118 (21.2)	439 (78.8)	.04
Summer	453 (20.0)	30 (6.6)	423 (93.4)		441 (20.0)	49 (11.1)	392 (88.9)		389 (19.4)	113 (29.0)	276 (71.0)	
Autumn	426 (18.8)	27 (6.3)	399 (93.7)		415 (18.8)	65 (15.7)	350 (84.3)		378 (18.8)	88 (23.3)	290 (76.7)	
Winter	762 (33.7)	52 (6.8)	710 (93.3)		743 (33.7)	86 (11.6)	657 (88.4)		685 (34.1)	158 (23.1)	527 (76.9)	

Abbreviations: IQR, interquartile range; ED, emergency department; CCI, Charlson Comorbidity Index; IRSAD, Index of Relative Socioeconomic Advantage and Disadvantage. ^a^Other: welfare institutions, prisons, mental and rehabilitation centres, private and rural hospitals.

**Table 3:** Logistic Regress Ion for Predictors of COPD-Related Readmission Within 30 Days and 90 Days

**Variables**	**30-Day Readmission ** **Adjusted OR (95% CI)**	**90-Day Readmission ** **Adjusted OR (95% CI)**
Age (y)	0.99 (0.98–1.01)	1.00 (0.99–1.02)
Male (vs female)	1.49 (1.06–2.09)	1.24 (0.95–1.61)
Indigenous (vs non-Indigenous)	2.47 (1.31–4.66)	1.52 (0.85–2.73)
Tasmanian region (vs South)		
North	1.25 (0.81–1.92)	0.82 (0.59–1.14)
North-West	1.80 (1.20–2.69)	1.21 (0.89–1.64)
Number of ED visits in previous 6 months		
COPD-related	1.48 (1.22–1.80)	1.52 (1.29–1.78)
Non-COPD-related	1.12 (1.03–1.23)	1.11 (1.03–1.21)

Abbreviations: OR, odd ratio; CI, confidence interval; ED, emergency department; COPD, chronic obstructive pulmonary disease.

###  Independent Predictors of Shorter Time to Readmission

The median time to the first COPD-related hospital readmission within 365 days was 75 days (interquartile range [IQR] 59–84). Seven variables were included in the multivariate Cox proportional hazard regression analysis (Table 4). Being Indigenous (median time to readmission 59 days [IQR 10–75], hazard ratio [HR]: 1.61, 95% CI: 1.10–2.37) and a higher number of COPD-related ED visits in the previous six months (median time to readmission 68 days [IQR 24–85], HR: 1.30, 95% CI: 1.21–1.39) were associated with shorter time to readmission within 365 days, while a higher CCI was associated with longer time to readmission (median time to readmission 81 days [IQR 39–165], HR: 0.91, 95% CI: 0.83–0.99).

**Table 4:** Cox Proportional Hazards Regression for Factors Associated With Time to Readmission Within 365 Days

**Variables**	**Unadjusted HR (95% CI)**	**Adjusted HR (95% CI)**
Age (y)	1.00 (0.99–1.01)	1.00 (0.99–1.01)
Male (vs female)	1.05 (0.88–1.26)	1.03 (0.86–1.24)
Australian born (vs oversea born)	0.99 (0.76–1.31)	
Indigenous (vs non-Indigenous)	**1.91 (1.34–2.74)**	**1.61 (1.10–2.37)**
Tasmanian region (vs South)		
North	0.88 (0.71–1.11)	
North-West	1.04 (0.84–1.29)	
CCI	**0.88 (0.81–0.96)**	**0.91 (0.83–0.99)**
Australian season of admission (vs winter)		
Spring	0.91 (0.71–1.15)	0.88 (0.69–1.11)
Summer	1.24 (0.97–1.58)	1.12 (0.88–1.44)
Autumn	1.03 (0.79–1.33)	1.02 (0.79–1.33)
Weekend admission (vs weekday)	0.95 (0.77–1.17)	
Number of ED visits in previous 6 months		
COPD-related	**1.35 (1.27–1.45)**	**1.30 (1.21–1.39)**
Non-COPD-related	1.04 (0.98–1.11)	
Length of index stay (days)	1.01 (1.00–1.02)	
Intensive care admission during index (vs no)	0.86 (0.56–1.33)	
Discharge destination (vs home)		
Nursing home	0.42 (0.16–1.13)	0.44 (0.16–1.17)
Other^a^	0.93 (0.66–1.32)	0.99 (0.70–1.41)

Abbreviations: HR, hazard ratio; CI, confidence interval; ED, emergency department; CCI, Charlson comorbidity Index; COPD, Chronic obstructive pulmonary disease. ^a^Other: welfare institutions, prisons, mental and rehabilitation centres, private and rural hospitals.

## Discussion

To our knowledge, this is the first Australian study to assess risk factors for COPD-related readmission. The rates of COPD-related readmission within 30 days (6.7%), 90 days (12.2%) and 365 days (23.7%) were comparable to those reported in studies from other countries, such as the United States,^19^ Spain^20^ and France.^21^ There are also studies that reported lower^22^ or higher^23^ rates.

 Patients who were male, Indigenous and living in a lower socieconomic region (North-West) were at significantly increased risk of 30-day COPD-related readmission. COPD-related and non-COPD related ED visits in the previous six months increased the risk of 30-day and 90-day readmission. Being Indigenous and having more COPD-related ED visits in the past six months were associated with shorter time to readmission, while higher CCI was related to longer time to readmission within 365 days.

Patients living in the North-West region of Tasmania were 80% more likely to be readmitted within 30 days compared to those living in the South. Several studies have demonstrated an increased risk of readmission for COPD among people living in deprived areas.^21,24^ The North-West region is Tasmania’s poorest region and, according to national data, people in lower socioeconomic areas are more likely to have poorer health status, higher smoking rates, poorer access to primary healthcare and are less able to pay for medication.^16,25^ A UK study found that disadvantaged communities have low ratios of general practitioners (GPs) per 1000 population.^26^ This finding is similar in Tasmania, with its lower number of full-time equivalent GPs in the North-West compared with Southern Tasmania (64.2 vs 78.7 per 100 000 population, respectively in 2012).^27^ The disparity in provision and access of primary healthcare services to the socioeconomically disadvantaged communities may explain their higher readmission risk.

Being Indigenous was the strongest risk factor for 30-day readmission, and significantly decreased the time to readmission within 365 days. National data indicate that Indigenous Australians are about three times more likely to be daily smokers, and have a 2.5 times higher prevalence of COPD and five times higher rate of hospitalisation for COPD.^28^

There have been conflicting results regarding the association of sex and COPD-related readmission in the literature. Zapatero et al^29^ reported male patients were 25% less likely to be readmitted, another study found no such association,^30^ while another demonstrated male patients were 45% more likely to be readmitted within 30 days.^31^ We found that male patients were 49% more likely to be readmitted within 30 days. A potential reason for this observation in Tasmania could be the higher rate of current male smokers (19.3%) compared to females (15.7%).^32^ There is also the possibility of more men having higher occupational exposure to dust and fume chemicals.^33^ Male patients may also be less likely to seek health advice during illness.^34^ This could delay management of mild/moderate symptoms before escalation to more severe symptoms requiring hospitalisation and then recurrent readmission.

The numbers of COPD-related and non-COPD-related ED visits in the six months prior to the index admission were associated with an increased risk of readmission within 30 and 90 days, as well as a significantly shorter time to COPD readmission. These findings are similar to prior studies that found a significant correlation between COPD readmission and previous ED visits.^24,30^ One explanation points to patients with a frequent exacerbation phenotype, who may be more likely to be readmitted.^35^ Patients with severe COPD may be more susceptible to frequent ED visits and admissions to hospital.^19,36^

Frequent ED visits, leading to hoispital admission, place an enormous burden on patients and the healthcare system. A previous study in Tasmania reported a 2.5 times higher per capita rate of ED visits in the North-West compared to the South.^37^ An Australian study that targeted older adults with complex health needs and disadvantaged socioeconomic communities demonstrated a significant reduction in the average number of ED visits via the creation of a comprehensive community care programme.^38^ There is a possibility that lack of community support frameworks that divert mild and moderate cases of COPD from hospital to settings in the community may increase the risk of readmission.

Our results show that patients with higher CCI had longer time to readmission within 365 days. Some studies have demonstrated that higher CCIs increase the risk of readmission,^29,36^ while another has shown a decreased risk of readmission with higher CCI.^39^ There is the possibility that COPD patients with numerous comorbidities may be consulting their GP more often, resulting in closer monitoring and longer time to COPD-related readmission. This also highlights the complexity of COPD which has been emphasised in the recent review of all-cause risk factors for readmission in COPD (comorbidities – heart failure, renal failure, depression).^40^ Hence a holistic approach which addresses these factors needs to be considered during the management of COPD.

The findings of this study are in line with the finding of Australain’s most recent national health report, which highlighted that social deterninants of health are contributing to inequalities in health between population groups.^41^ It also demonstrates the need for comprehensive strategies to be considered in national management guidelines,^42^ to provide more equitable COPD management and improved patient outcomes.

 The present study has the following public health implications. It has identified that male patients, Indigenous people, patients with recent ED visits, and those living in the lower socioeconomic North-West area of Tasmania are at increased risk of COPD-related readmission. Future studies focused on improving patient-centred access to healthcare within the community, especially in the lower socioeconomic areas, may improve health outcomes, and reduce ED visits and hospital readmissions.

 The main limitation of this study is the relatively low number of patients and the inherent shortcomings related to the retrospective design using administrative data, such as missing and incomplete data. However, missing and incomplete data accounted for only 1.5% of the dataset. Further, the low numbers are a reflection of our small state rather than an incomplete capture of patients; using the National Minimum Dataset data, all patients with an overnight admission due do COPD between 2011 and 2015 were able to be captured. There is also the possibility of inacurate or inconsistent coding, the extent of which is unknown. Futhermore, individual smoking status and several clinical factors (eg, previous exacerbations of COPD, the severity of the disease, decline in lung function, etc) were not available from the data source.

 Despite the limitations, our study is the first to investigate COPD-related readmission in Australia and has identified the prevalence and key risk factors for readmissions due to COPD. This five-year logitudinal study comprised all patients admitted with COPD to the four main public hospitals in Tasmania with minimal interstate hospital visits. Further studies are required to assess clinical risk factors, such as lung function and smoking status, that are not available in the National Minimum Dataset for a broader understanding of determinants of COPD-related readmissions.

## Conclusion

 The prevalence of 30-day, 90-day and 12-month COPD-related readmissions in Tasmania were 6.7%, 12.2% and 23.7%, respectively. Being male, Indigenous and living in the North-West region were significant predictors of COPD-related readmission. Previous ED visits were associated with increased risk of 30-day and 90-day COPD-related readmissions and shorter time to readmission within 365 days. Studies on interventions aimed at providing and improving access to community-based healthcare services to males, Indigenous people and those living in disadvantaged socioeconomic areas may reduce COPD-related readmissions.

## Ethical issues

 The study was approved by the Tasmania Human Research Ethics Committee (H0017433).

## Competing interests

 Authors declare that they have no competing interests.

## Authors’ contributions

 Conceptualisation: CN, BB, BW, GP, and LK. Methods: CN, BB, BW, GP, and LK. Formal analysis: CN, BB, BW, GP, and LK. Investigation: CN, BB, BW, GP, and LK. Manuscript draft preparation: CN. Manuscript review and editing: CN, BB, BW, GP, and LK. Supervision: BB, BW, GP, and LK. Project administration: CN.

## Funding

 This research did not receive any specific grant from funding agencies in the public, commercial, or not-for-profit sectors. CN was supported by an Australian Government Research Training Program (RTP) Stipend and RTP Fee-offset Scholarship through the University of Tasmania.
